# Knee Osteoarthritis: Kinesiophobia and Isometric Strength of Quadriceps in Women

**DOI:** 10.1155/2022/1466478

**Published:** 2022-05-05

**Authors:** Saulo Machado, Érika Santana, Victor Brito, Leonardo Maciel, Lucindo J. Quintans Júnior, Walderi da Silva Junior, Jader de Farias Neto, Henrique Douglas Melo Coutinho, Bonglee Kim, Valter J. de Santana Filho

**Affiliations:** ^1^Programa de Pós Graduação Em Ciências da Saúde, Universidade Federal de Sergipe, São Cristóvão, Sergipe, Brazil; ^2^Graduado Em Fisioterapia, Universidade Federal de Sergipe, São Cristóvão, Sergipe, Brazil; ^3^Departamento de Fisioterapia, Universidade Federal de Sergipe, Campus Lagarto, São Cristóvão, Sergipe, Brazil; ^4^Departamento de Farmácia, Universidade Federal de Sergipe, São Cristóvão, Sergipe, Brazil; ^5^Departamento de Fisioterapia, Universidade Federal de Sergipe, Campus São Cristovão, São Cristóvão, Sergipe, Brazil; ^6^Regional University of Cariri, URCA, Crato, Brazil; ^7^Department of Pathology, College of Korean Medicine, Kyung Hee University, Seoul 02447, Republic of Korea; ^8^Korean Medicine-Based Drug Repositioning Cancer Research Center, College of Korean Medicine, Kyung Hee University, Seoul 02447, Republic of Korea

## Abstract

**Introduction:**

Osteoarthritis is a disease characterized by progressive wear and tear of the joint, with the knee being the most affected region. These patients have reduced mobility and mobility, among other symptoms. Thus, it is necessary to know the variables that influence the ability to walk.

**Objective:**

To analyze how much the gait capacity, in the performance of the six-minute walk test, can be influenced by the maximum isometric strength of the quadriceps or by kinesiophobia in women with knee osteoarthritis.

**Materials and Methods:**

This is a cross-sectional study with a sample of 49 women diagnosed with osteoarthritis. The evaluation was carried out in a single moment. Variables studied isometric quadriceps strength, level of fear of movement (kinesiophobia), and ability to walk. Simple linear regression analyzes were performed, with gait ability as the dependent variable and maximum isometric strength and kinesiophobia as independent. Data were presented with mean and standard deviation and were analyzed by the SPSS Statistic 22.0 software, considering *p* < 0.05 as significant.

**Results:**

The maximum isometric strength presents a significant difference, directly interfering with the gait ability; as kinesiophobia does not show a statistically significant difference, it does not directly interfere with the ability to walk.

**Conclusion:**

Maximal quadriceps isometric strength directly interferes with gait ability in women with knee osteoarthritis, thus suggesting the inclusion of this strategy in treatment programs for this population.

## 1. Introduction

Knee osteoarthritis is characterized by progressive wear or degeneration of anatomical structures, especially the menisci and cartilage, being the main cause of pain and locomotor disability worldwide [1].

Epidemiologically, it has a higher incidence in the population over 75 years of age, in addition to affecting mainly women, with about 50% of these people showing symptoms such as loss of strength and muscle mass, morning joint stiffness, bone crackling, pain, and mainly reduced gait ability, which can be influenced by several factors, in addition to radiographic clinical signs, narrowing intraarticular space, osteophytes, subchondral bone sclerosis, and cystic formations [2].

This disease is classified by Ahlback (1968) [3] and modified by Keyes and Goodfellow (1992) [4] in five degrees: degree 1, those with reduced joint space; grade 2, those that present space obliteration; grade 3, those with wear of the tibial plateau <5 mm; grade 4, with wear of the tibial plateau between 5 mm and 10 mm; and grade 5, those with severe subluxation of the tibiofemoral joint; however, in many cases, the symptoms are not directly related to the degree observed in the X-ray examination. The symptoms are believed to have a strong association with genetic, environmental, and age factors; the knee joint being more prone to its appearance due to the great overload imposed on this structure daily [1].

Some factors can influence the onset or progression of OA, such as inflammatory processes located in the affected joint or even systemic inflammation factors that, in the long term, negatively interfere with global muscle strength [5]. Mechanical factors interact in an important way in the onset and progression of OA, such as the reduction in muscle volume, in the anatomical cross-sectional area, and in the length of the muscle fascicles. However, joint changes are directly related to joint geometry and load distribution. The mechanical misalignment of the tibia and femur causes the overload on to be incorrectly distributed in the joint, reducing the density of the articular cartilage and causing a decrease in the intraarticular space. All these changes led to a reduction in the performance of activities of daily living and simple tasks such as walking; these conclusions were reached by Frason [6].

Therefore, it is necessary to understand the main factors that cause the reduction of gait capacity, thus reducing the mobility and independence of these individuals. In this context, the six-minute walk test, used to assess aerobic capacity, can also be interpreted as an assessment of gait capacity for individuals with musculoskeletal changes in the lower limbs. This test may be related to changes in physical function, as a greater distance covered will mean a better capacity [7]. In this patient profile, some variables can influence gait performance and two stand out, muscle strength and kinesiophobia, the fear of performing the movement.

Among these variables, it is already known that muscle strength undergoes an important reduction in people who develop OA. Of the different characteristics that strength presents, in this profile of patients, maximum isometric strength is the one that suffers the most negative influence [8]. Both the extensor and flexor muscles are important shock absorbers that help to stabilize the joint, yet the dynamic stability of the knee joint is dependent on maintaining the muscle strength of these muscles [9]. In the context of knee osteoarthritis, the extensor muscles play a more stabilizing role [10]. Studies demonstrate that quadriceps weakness has been associated with knee osteoarthritis on radiographic examinations without a history of knee pain and that decreased quadriceps muscle strength was clearly associated with increased disability in people with osteoarthritis [11].

In addition to this symptom, environmental factors and people can interfere with other symptoms of patients with knee osteoarthritis, such as kinesiophobia. This can be defined as excessive, irrational, and debilitating fear of movement and physical activity, which results in feelings of vulnerability to pain or fear of recurrence injury [12]. Thus, there is a need to assess this interference from fear of movement associated with pathological processes; for this, it is necessary to use clinical assessment tools to verify fear related to pain, and it can help to reduce the factors previously mentioned by Vitsarut [13]. To assess fear when moving, Kori [14] developed the original Tampa Scale for Kinesiophobia (TSK), of 17 items from a pain-related questionnaire to measure fear of moving in patients with chronic pain, which was adapted for several languages such as Portuguese [13]. Thus, the Tampa Scale of Kinesiophobia (TSK) was formed.

The possible direct relationship of these variables with gait ability is necessary for a better adaptation of the rehabilitation process of these individuals. Therefore, this project is justified by the need to analyze the existence of correlation between these variables, to be added to the service protocols for improving these variables.

Understanding the importance of these variables for individuals with knee osteoarthritis, the aim of this study was to analyze the influence of kinesiophobia and maximal isometric muscle strength of the quadriceps on the performance of the six-minute walk test in patients with knee osteoarthritis.

## 2. Materials and Methods

### 2.1. Study Design

This is a cross-sectional study, where gait ability, isometric quadriceps strength, and kinesiophobia were evaluated in women with knee osteoarthritis.

This project has already been approved by the Ethics Committee in Research with Human Beings of the Federal University of Sergipe (UFS) (CAAE no.: 64810117.0.0000.5546, approval no.: 1.961.364).

### 2.2. Participants

The sample consisted of 49 women, the amount of the sample being for convenience, among those followed by the Orthopedics and Traumatology Outpatient Clinic of the University Hospital of the Federal University of Sergipe (UFS), with clinical diagnosis of knee osteoarthritis, between grades 2 and 4, according to Ahlback [3, 4], without surgical indication, performed by an orthopedist. Those individuals who accepted to participate voluntarily signed the free and informed consent term according to the norms expressed in the resolution 466 of the national health council.

The inclusion criteria were women, age between 30 and 80 years, who have pain above 3, on a scale of 0–10, preserved cognitive functions, and availability to carry out the program.

The exclusion or discontinuation criteria were those who did not agree with informed consent, who performed previous surgical procedures on the knee or current indication for it, did not perform the assessment or the protocol, and those who felt pain in any segment other than knee pain.

### 2.3. Study Design

#### 2.3.1. Evaluation

Initially, the participants were evaluated by the orthopedist to verify their eligibility to research. Subsequently, the participants filled out the evaluation form, containing their epidemiological and sociodemographic data, and accepted to participate, agreeing with the free and informed consent form. Subsequently, the evaluation protocol was performed.

#### 2.3.2. Studied Variables

Aerobic and gait capacity: performed through the six-minute walk test (6MWT), it is a simple test and easy to apply, where you can observe some factors that influence the gait of patients. This form of assessment has validity and good reliability in patients with cardiorespiratory changes and can be applied and also has good validity and reliability in patients with chronic changes, including elderly women with knee osteoarthritis [15–17]. Its performance takes place in a corridor marked every 1 meter, totaling 30 meters in length, the individual walked the path, being instructed to maintain the maximum speed, as constant as possible, for 6 minutes, performing the greatest number of turns in the corridor. Until the end of times, the individual was instructed to verbalize the discomfort, as well as the possibility of interrupting the test at any time. At the end, the number of meters walked by each subject was recorded [15].

Kinesiophobia: assessed using the Tampa Scale for Kinesiophobia (TKS), patients' fear of movement was investigated. The Tampa scale has been used in different populations with chronic disorders, such as low back pain, and it can transpose reliability and validity, with adequate internal consistency, to investigation in other joints such as the knee [17]. The scale consists of 17 items that address pain and the intensity of symptoms, scores range from 1 to 4, the maximum obtained is 68 points, and the minimum is 17 points. According to the scale score, 1 point “strongly disagree,” 2 points “partially disagree,” 3 points “partially agree,” and 4 points “strongly agree.” Higher scores indicated greater kinesiophobia [18, 19].

Isometric quadriceps muscle strength: it was performed using a load cell, connected to the signal analysis device. This form of assessment is considered the gold standard for assessing maximal isometric strength. Its validity and reliability have already occurred for alterations in the knee joint; in addition, the protocol for performing the test and its positioning were also included in the validity and reliability assessments [20]. For this study, version 1.8.1 of the Chronoiump Boscosystem (Force Sensor Kit, Spain) was used and placed on an extension chair commonly used for strengthening exercises, where the individual sits, with knees at 90° and hips at 110°, thus ensuring the highest torque of the evaluated muscle (quadriceps), where the individual was asked to perform as much force as possible for the knee extension movement, holding for 10 seconds.

### 2.4. Statistical Analysis

The data were presented using mean and standard deviation. For normality analysis, the Shapiro–Wilk test was used. In the context of knee osteoarthritis, the extensor muscles play a more stabilizing role. For the variable dynamometry, the values found in the right and left lower limbs were summed. A partial correlation test was also performed, considering age as a covariate. Simple linear regression analysis was performed with kinesiophobia as the dependent variable and dynamometry as an independent variable. Statistical analyzes were performed using the SPSS Statistic 22.0 software, with *p* values considered significant when less than 0.05.

## 3. Results

A total of 49 women diagnosed with knee osteoarthritis participated in the research.  Table 1 provides the characterization of the sample, where the importance achieved by the normality test is also demonstrated or evaluated, thus demonstrating the homogeneity of the population, since there was no difference in the characterization variables.

Table 2 provides the values found in the evaluation of kinesiophobia and maximum isometric strength of quadriceps through the dynamometer, presented by mean and standard deviation.

Table 3 provides the values found in the analysis of simple linear regression. For this analysis, walking ability was taken as a dependent variable, assessed through the six-minute walk test, and as independent variables kinesiophobia and maximum isometric strength of quadriceps.

As can be seen, the independent variable kinesiophobia did not present a significant statistic, showing that it does not directly affect walking ability, different from the maximum isometric force that showed a significant difference, demonstrating that there is a direct interference in the gait capacity.

The *R*^2^ must be interpreted as a percentage showing that various variables can interfere with the ax capacity, the maximum isometric strength represents 27%, and as the beta value of the independent variable was given positively, it represents that the relationship is direct, that is, how much the greater the isometric force, the greater the walking capacity.

## 4. Discussion

The present study aimed to verify if there is a relationship between gait ability, kinesiophobia, and maximal isometric strength, and its data showed a direct relationship between the variables maximal isometric strength (MIS) of the quadriceps (*p*=0.01^*∗*^) and ability to gait in women with knee osteoarthritis (*R*^2^ = 0.27). This can be seen through the significance values found when a linear regression is performed between the maximum isometric strength score and walking capacity. These results can help decision making in clinical practice in order to generate greater stimuli to gain independence and gait ability, in addition to serving as a guide for future studies that influence these variables. We indicate as a limitation of the study the sample size, which, even being a homogeneous sample, would have a greater representation with more women included.

Knee osteoarthritis can negatively influence the ability to walk through some changes, for example, mechanical changes in the lower limbs. In moments of gait, especially the stance phase, when there is a reaction force to the ground, the leg can make an excessive adduction movement, inhibiting some musculatures and causing pain [21]. Other changes in March seen in people with knee OA include reduced walking speed, shortened stride length, reduced pelvic rotation, and lateral trunk tilt; these were cited by Van der Esch, 2011 [22].

A study mentions that the decrease in quadriceps strength is around 30–50% in individuals with OA, when compared to healthy individuals within the same age group [23]. They also suggest an imbalance of muscle strength between flexors and extensors, and in general, the decrease in quadriceps strength occurs more sharply than that of the hamstrings. This imbalance of forces can cause functional changes that accelerate joint degeneration and/or cause functional disability [24].

The six-minute walk test, in addition to assessing aerobic capacity, can also be interpreted as an assessment of gait capacity for individuals who have musculoskeletal changes in the lower limbs; this test can be related to changes in physical function, since the greater the distance traveled, the better its capacity, according to Duarte (2013) [7].

In addition to the quadriceps ratio, it is observed that the lateral inclination reduces the demand on the hip abductor muscles, potentially leading to the weakening of the abductor over time. The gluteus medius is the main abductor of the hip and a large portion of this muscle acts in the frontal plane to stabilize the pelvis and lower leg during march. An increase in lateral trunk tilt is often used by participants to relieve the medial knee [24]. In this context, the understanding of strength reduction, especially isometric, can generate benefits for these patients, in association with other exercise treatment protocols [25].

The aforementioned theoretical framework corroborates the findings of the present study, as women who presented greater quadriceps isometric muscle strength obtained a better score in the six-minute walk test, thus showing better gait capacity, proving that the direct relationship between the variables is clinically effective [26]. In addition to the inclusion of strength training, as previously suggested, other treatment modalities can be associated as an adjunct in the treatment of these patients, such as the use of orthoses and electrotherapeutic means [27, 28].

Kinesiophobia is a poorly adaptive strategy that leads to avoiding physical activity because of fear related to pain [14]. The experience of pain can create a vicious cycle in which there are cognitive and behavioral disorders in an amplified way. The present study demonstrated that the values of gait ability and kinesiophobia are inversely proportional, since the value of B1 was negative, but did not present a statistically significant value (*p*=0.39), demonstrating that this interference was not related to gait strong.

Doury-Panchout et al. in 2015 reported in their studies with knee osteoarthritis patients that the distance covered in meters during the six-minute walk test was significantly greater for patients without kinesiophobia than patients with kinesiophobia [29]. This does not corroborate with the statistical findings of our study, where a negative B^1^ value (*B*^1^ = -2.18) was shown, explaining that the relationship is not direct, so it is recommended to carry out more studies with good rigor, methodologically associating these variables to examine this relationship more adequately. In addition, they confirmed that avoiding activities increases disability and can increase the risk of medical complications, such as obesity and diabetes [30, 31].

## 5. Conclusions

In summary, the study showed that isometric quadriceps strength directly interferes with the gait ability of women with knee osteoarthritis. Simple regression indicated that the independent variable kinesiophobia did not present significant statistics, showing that it does not directly interfere with gait ability; different from the maximum isometric strength, there was a significant difference, directly interfering with the gait ability. This finding can be used as a basis to guide the treatment of women with hip osteoarthritis, for which we suggest the inclusion or intensification of the strengthening of the knee extensor muscles, especially in an isometric way, with the purpose of improving one of the largest complaints of this population, the ability to move, to walk.

## Figures and Tables

**Table 1:** Sample characterization.

Variables	Mean (SD)	*P*
Age (years)	58.49 (9.43)	0.23
Weight (kg)	78.93 (13.26)	0.76
Height (m)	1.55 (0.07)	0.24
BMI (Kg/m^2^)	32.6 (6.33)	0.48

The data are presented in mean and standard deviation and the p values are representing the values of the normality test: Shapiro-Wilk. SD, standard deviation (n = 49).

**Table 2:** Result of the evaluation of kinesiophobia and maximum isometric strength.

Variables	Mean (SD)
Walking capacity	338.12 (98.64)
Kinesiophobia	48.46 (5.67)
Maximum isometric force	37.32 (18.26)

SD, standard deviation.

**Table 3:** Result of the simple linear regression analysis of the evaluation of kinesiophobia with MIS.

Dependent variable	Independent variable	*R* ^2^	B0	B1	*P*
Walking capacity	Kinesiophobia	0.16	443.74	−2.18	0.39
	MIS	0.27	266.42	1.92	0.01^*∗*^

A partial correlation with age was performed as a covariate. MIS, maximum isometric force; B0, beta of the constant; B1, beta of the independent variable. ^*∗*^Significance *p* < 0.05.

## Data Availability

The data used to support the findings of this study are available from the corresponding author upon request.
